# High nest failure in a zebra finch population and persistent nest predation by a monitor lizard

**DOI:** 10.1002/ece3.11281

**Published:** 2024-04-15

**Authors:** Marc Naguib, Evelien ter Avest, Chris Tyson, Martin J. Whiting, Simon C. Griffith, Hugo Loning

**Affiliations:** ^1^ Behavioural Ecology Group Wageningen University & Research Wageningen The Netherlands; ^2^ School of Natural Sciences Macquarie University Sydney New South Wales Australia; ^3^ School of Biological, Earth and Environmental Sciences University of New South Wales Sydney New South Wales Australia

**Keywords:** nest predation, predation, predator cognition, sand goanna, *Taeniopygia castanosis*, *Varanus gouldii*

## Abstract

Predation is well known to have substantial effects on behaviour and fitness in many animals. In songbirds, nest predation is rarely observed directly, so that research focusses primarily on the consequences of predation and less on the behaviour of the predator. Here, we report predation data in a zebra finch (*Taeniopygia catanosis*) nest box population, highlighting a 22‐min‐long sequence, captured on video, of a sand goanna (*Varanus gouldii*) predating a zebra finch nest in the wild. This monitor lizard appeared to be extremely persistent with climbing and jumping up to the next box nine times, including three successive unsuccessful attempts that lead to a change in approach strategy. It removed all six nestlings from the nest box during those repeated approaches and consumed them. In combination with overall high predation rates in the study population we document here, the findings highlight the role that a single predator species can have on nest success and, thus potentially also breeding decisions and social organisation of the prey population. Specifically so in a species like the zebra finch which synchronises reproductive attempts through the use of social information acquired through nest inspections and which uses social hotspots where they could gather information on changes in local social composition due to the individualised signals they use.

## Graphical abstract

Predation is well known to have substantial effects on behaviour and fitness in many animals. Here we report predation data in a zebra finch (*Taeniopygia castanosis*) nest box population, highlighting a 22‐min‐long sequence, captured on video, of a monitor lizard predating nine times in a row on a zebra finch nest in the wild. The findings highlight the role even single predators can have and stress that predation could have a strong impact on breeding decisions and social organisation.

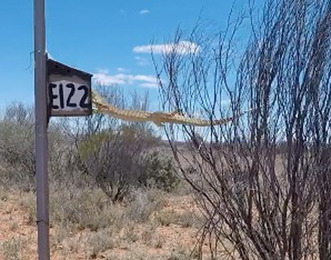

## 1 INTRODUCTION

In arid habitats, seasonal reproductive success in birds can vary significantly because of the unpredictable frequency of rainfall (Morton et al., 2011). In the case of zebra finches (*Taeniopygia castanosis*), rainfall is essential for the growth and seed production of a wide variety of grasses. These are necessary for providing essential energy stores for females to produce eggs and subsequently, for the parents to provision their young (Funghi et al., 2020). However, breeding decisions may also be a consequence of predation pressure on either eggs or nestlings (Griffith et al., 2008), and nest predation can be above 65% of nests (Zann, 1994). In some systems, predators can have a major impact on population breeding success and persistence (Fulton, 2019; Martin, 1995; Savidge, 1987; Suhonen et al., 1994). Nesting birds provide an excellent opportunity to monitor seasonal breeding success because nests can easily be monitored and nest boxes can be deployed to facilitate monitoring if the target species readily uses them for breeding. Furthermore, the use of nest boxes can drastically decrease predation pressure and the establishment of nest boxes for zebra finches reduced predation to 2% of nests (Griffith et al., 2008). Yet, in general, we have a relatively poor understanding of the behaviour and impacts of predators on nesting birds.

Sand goannas (*Varanus gouldii*) are terrestrial, active foragers that rely heavily on vomerolfaction (chemosensory searching) to locate prey (Cooper, 2007). They have strong forelimbs and well‐developed claws with which to excavate prey from below ground although they will occasionally climb trees to capture prey. Their diet is extremely broad and includes orthopterans, beetles, centipedes, roaches, spiders, scorpions, ants, phasmids, cicadas, insect larvae, birds (including nestlings), lizards, snakes, fishes, small mammals and reptile eggs (mainly lizard and crocodile) (Godwin et al., 2020; Losos & Greene, 1988; Pianka, 1986). Although we have a relatively good understanding of their diet based on preserved museum material, we have few observations of hunting behaviour and prey capture in the wild (Godwin et al., 2020). Overall, sand goannas are medium to large‐sized monitor lizards (up to 1.6 m) broadly distributed across Australia (Cogger, 2018). Although they occur in a variety of habitats (e.g. chenopod shrubland, eucalypt woodlands, sandplains, sandy deserts), they are typically found on sandy substrates where they take refuge in burrows (Dryden et al., 2004). Given their diet and ability to climb, they are a potential predator of zebra finch eggs and nestlings (Immelmann, 1962).

Here, we report a detailed predation sequence, captured on film, of a sand goanna predating six zebra finch nestlings from a single nest box in the arid zone of western New South Wales, Australia. We also report data on egg and nestling predation in a breeding season for a population of zebra finches. This documentation highlights not only the persistency of predators, but such events also help shape our view on how predation can affect fitness and potentially also population‐wide breeding decisions and social organisation.

## 2 METHODS

The extremely persistent sequence of predation attempts by a sand goanna on a zebra finch nest we are reporting here, took place in a nest box at our zebra finch study site on 16 October 2023 at Gap Hills, UNSW Arid Zone Research Station Fowlers Gap, New South Wales, Australia (30.9490° S, 141.7675° E). To determine parental nest visits and to monitor possible predation, we filmed this nest box, along with others, for over 7.5 h using a GoPro 5, capturing the predation sequence at 13:00 in the afternoon. The nest box was one of 180 nest boxes at Gap Hills. We have worked on the zebra finches at this site for 19 years without any significant nest predation, particularly on zebra finch nests inside nest boxes (Griffith et al., 2008). However, this spring predation rates were high. This spring, sand goannas also appeared to be more abundant in the area, with relatively more anecdotal sightings by fieldworkers relative to previous years, perhaps due to good ecological conditions over the past few years (Loning, Verkade, et al., 2023).

## 3 RESULTS AND DISCUSSION

**FIGURE 1:**
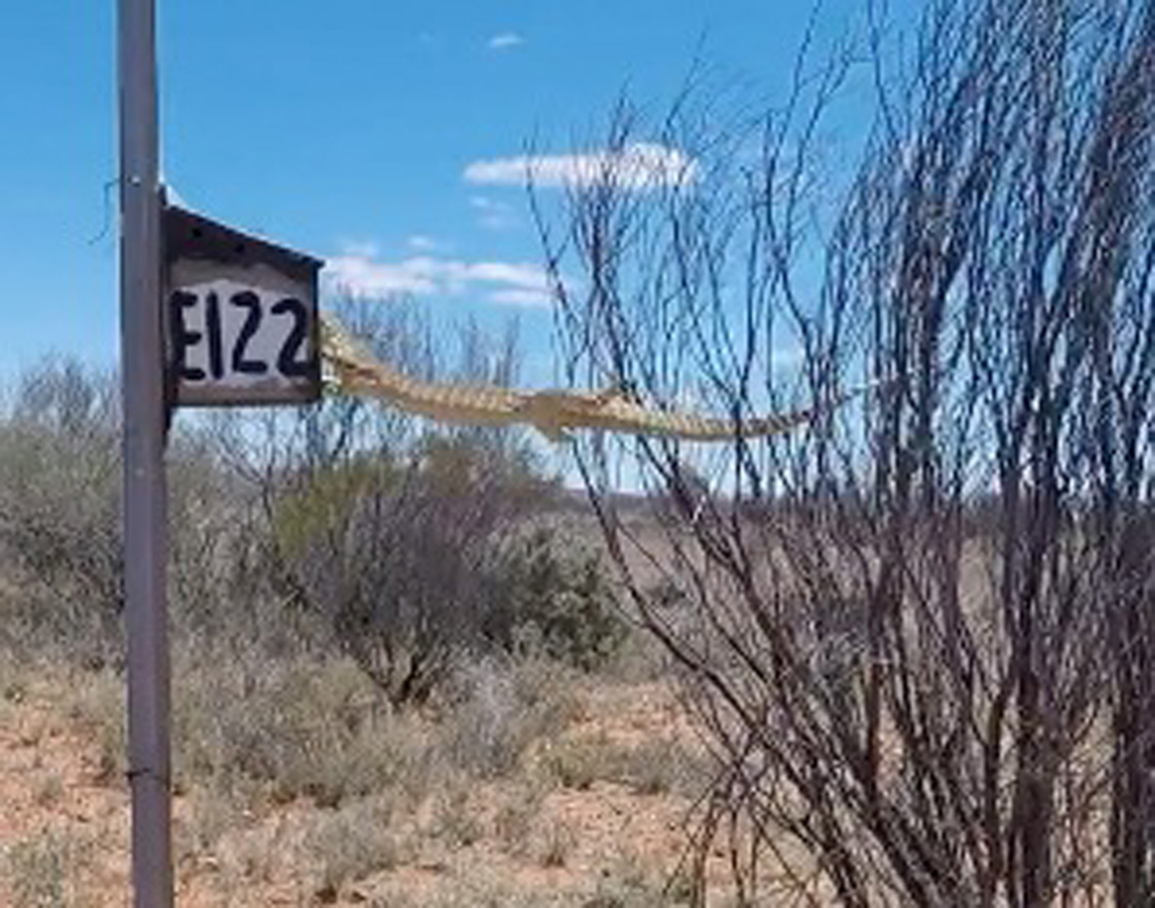
Screenshot of the sand goanna jumping from the bush to the nest box. The individual measured approximately 38 cm (SVL) with a total length of 75 cm (estimated from measurements of structural features around the nest box; Figure 4). The bottom of the nest box was positioned 111 cm from the ground and approximately 42 cm away from the nearest part of the adjacent bush (Figure 4). Video/photo‐screenshot credit: Marc Naguib.

The sand goanna made nine attempts to reach the nest box by first climbing an adjacent bush and jumping onto the nest box and eventually also by climbing up the steel post on which the box was attached. The complete predation sequence lasted 22 min and consisted of multiple sequential visit attempts, some unsuccessful, to the nest box using different strategies and consumption of all six nestlings that were in the nest box. This predation sequence is striking for three reasons (1) multiple arboreal jumps by a typically terrestrial lizard, (2) the persistent efforts to access the nest box despite several failed attempts and (3) the switch of strategy from climbing the bush and then jumping, to directly climbing the star picket post on which the nest box was mounted. The video had been recording for a total of 03 h:05 m before the goanna first appeared in the video. Therefore, it appears that it arrived at this active nest when first appearing in frame and that we captured the entire event. The sand goanna initially, without apparent hesitation, climbed the bush and then jumped onto the nest box (Figure 1), subsequently reaching into the nest box twice. This suggests that the sand goanna expected a nest in this nest box, either through previous visits, strong clearly localisable chemical cues emerging from the nest box, having developed a search image that these nest boxes contain food or a combination of these factors. On the first occasion, it removed a single nestling, which it consumed while hanging onto the nest box, whereas on the second occasion, it removed two additional nestlings and jumped down to eat them. Afterwards, the sand goanna climbed the bush again, jumped across to the nest box and consumed another nestling (fourth nestling). After again dropping to the ground to eat the nestling, it climbed through the bush and jumped to the nest box, taking two more nestlings in succession (fifth and sixth nestling), which were eaten while hanging off the nest box, and on the ground respectively. The nest box was empty at this point. Nevertheless, the sand goanna made three more attempts to access the nest box by climbing the bush and jumping towards the nest box. On all three occasions, it fell short and dropped to the ground. Finally, it climbed the nest box pole and accessed the nest box entrance by sitting on the nest box, rather than hanging on the entrance point at the front of the nest box. Since it had previously already eaten all six nestlings, this final attempt appeared to be a last check of whether any nestlings remained in the nest box. The sand goanna dropped to the ground one final time and could be seen walking directly away from the nest box 22 min after first appearing on camera. The video continued to run for another 04 h:11 m during which no sand goanna was seen, indicating that it did not return in the short term.

**FIGURE 2:**
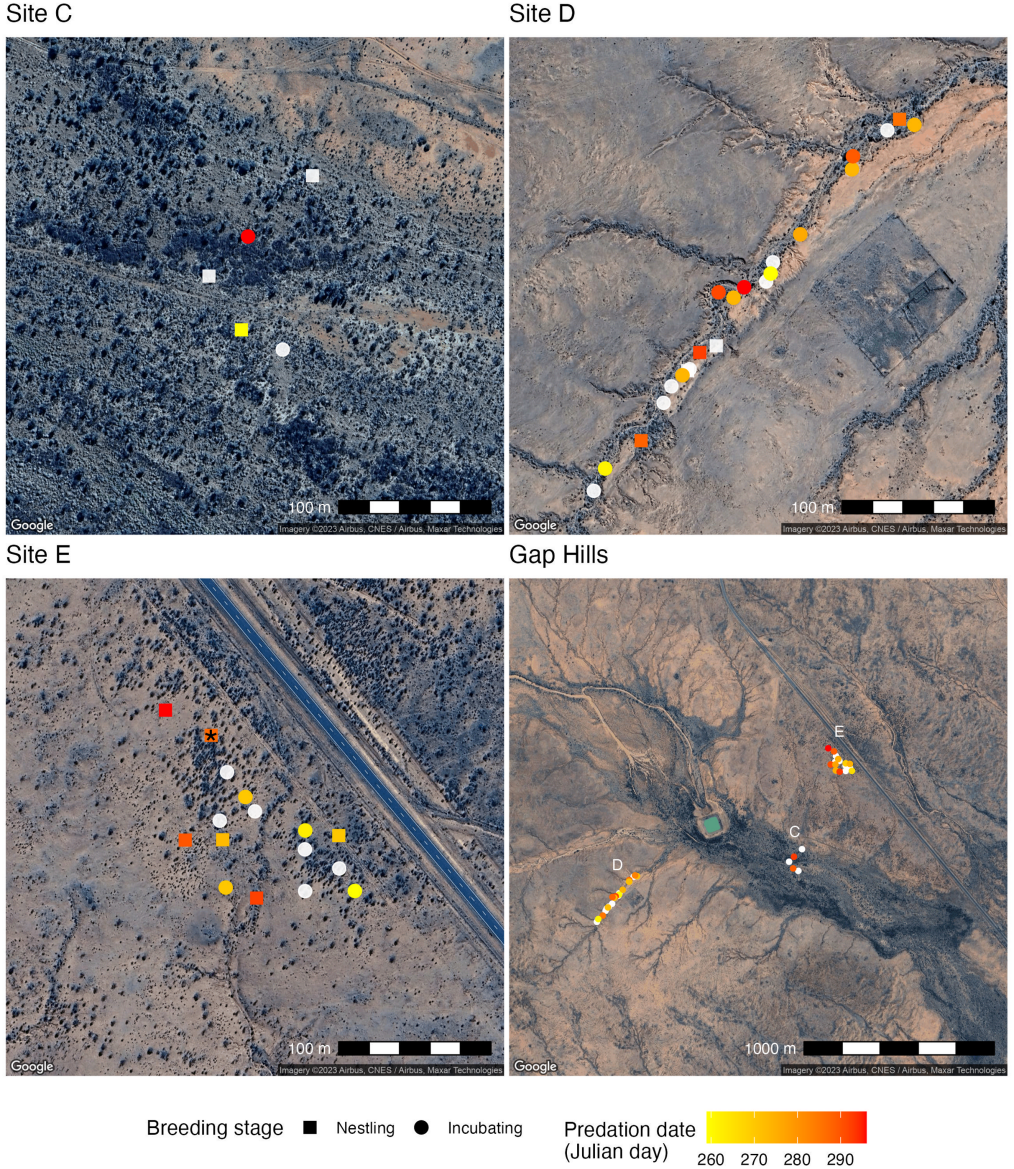
Predation events at three main sites (C, D, E) within the overall study area (Gap Hills). Symbols represent nest boxes with breeding events, and colours indicate time of predation; white, non‐predated nests with breeding activity. The nest box with the documented sand goanna predation is indicated in Site E by a star.

**FIGURE 3:**
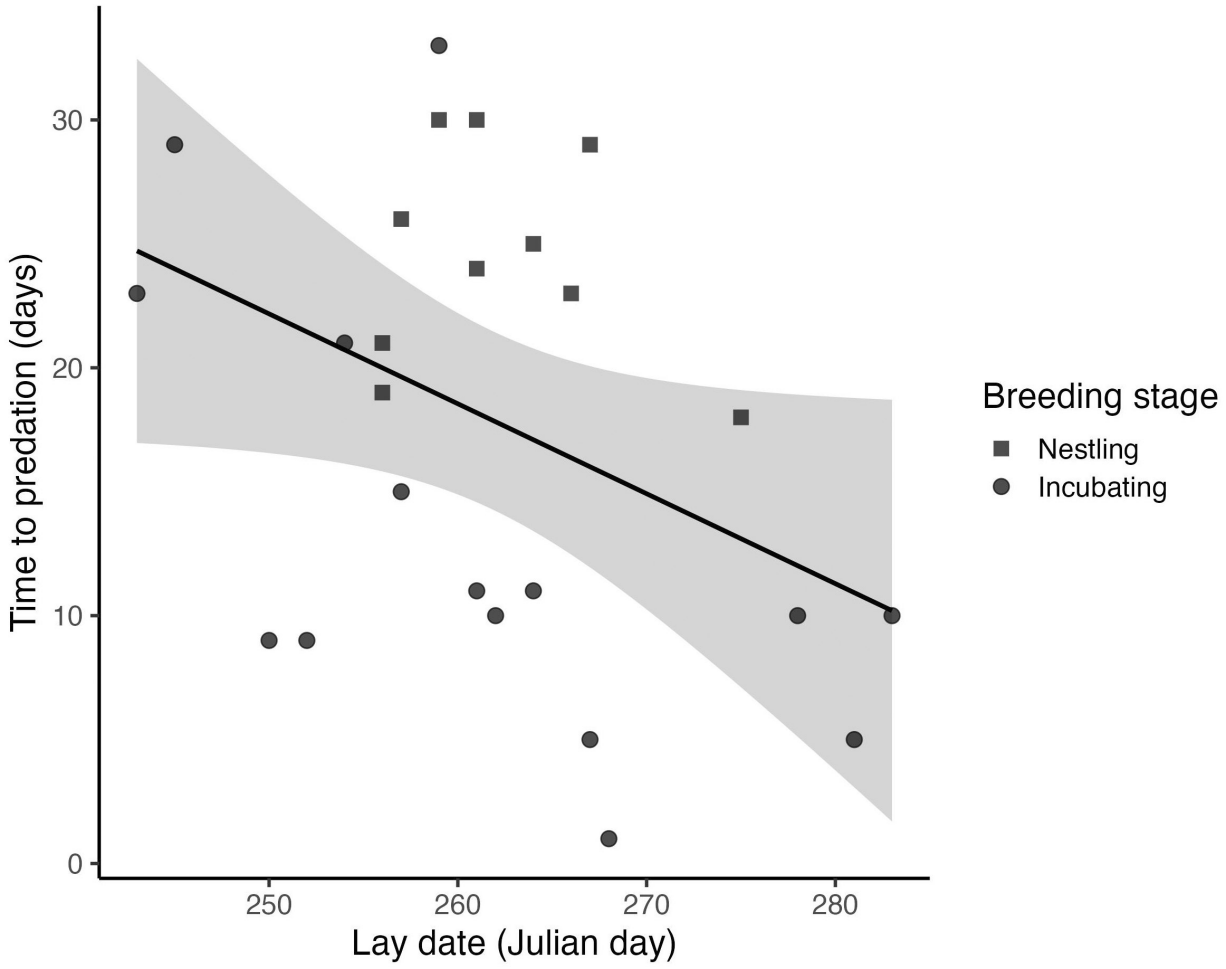
Time to predation in days of zebra finch nests relative to lay date and breeding stage. Nests that started later in the breeding season tended to be predated more quickly than nests which began earlier in the breeding season (*F*_1,23_ = −4.24, *p* = .0509, see text). The model regression line is shown in black and the 95% confidence interval is shown in grey.

**FIGURE 4:**
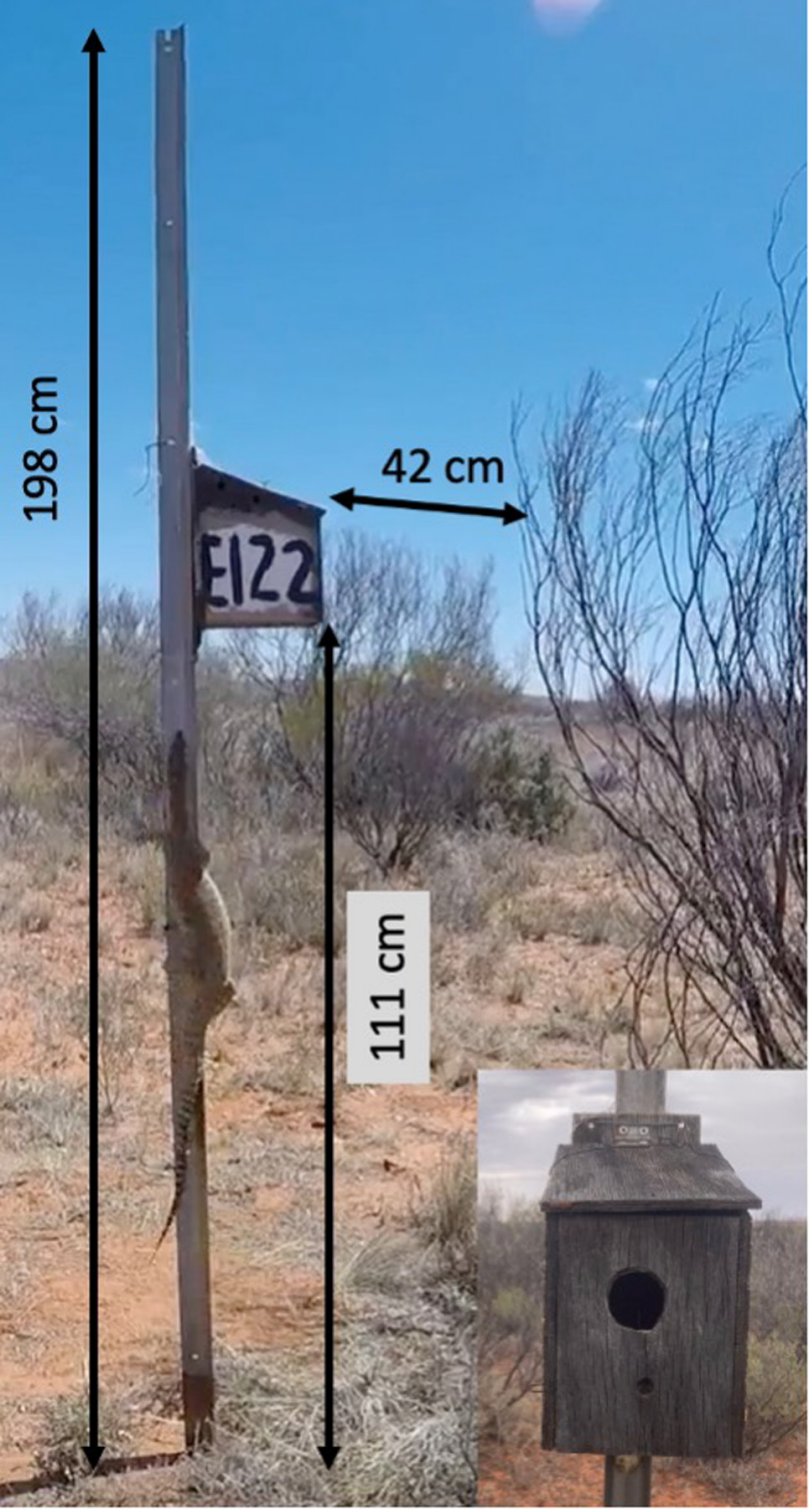
Screenshot from the video of the sand goanna climbing the nest box pole with arrows indicating distances, as measured directly at the site using a tape measure. Bottom right inset photograph of the front of this nest box with the diameter of the entrance hole being 33 mm (photo‐screenshot credit: Marc Naguib; insert photo credit Frigg Speelman).

Noteworthy beyond this documentation is that multiple nest boxes in this area suffered the same fate, which differed strikingly from previous years as nest boxes had very low predation rates and were shown to have significantly lower predation rate than natural nests (Griffith et al., 2008). We assume that many of the predated nests reported here also were predated by a sand goanna, possibly the same individual, but across our study population, predation of nestlings is likely from multiple individuals. From mid‐September to the end of October 2023, we documented 15 cases of egg predation and nine cases of nestling predation, representing 70% of the total zebra finch nesting attempts in this population over that time (Figure 2), with late breeders tending to suffer the most predation (*F*_1,23_ = −4.24, *p* = .0509, linear model, R version 4.3.3, Figure 3). Unfortunately, we were unable to confirm the identity of the predator of these other nests. Nonetheless, our observations of the hunting behaviour of this one individual, coupled with increased sightings of sand goannas compared to previous years when predation of zebra finches was much lower, raises the possibility that sand goannas are significant predators of zebra finch nestlings also in natural nests and highly efficient at finding active nests with young to predate. The immediately neighbouring nest box a few meters away was predated a few days later, when nestlings also were 12 days old, and nest remains on the ground appeared similar to the event reported here, suggesting that it was the same individual. Indeed, these nest boxes likely have distinct chemical signatures that could attract sand goannas. However, because nest boxes are distinct in appearance, it also is quite possible that the sand goannas may be learning a search image. This warrants further investigation but would imply that use of nest boxes in research and conservation needs to be monitored regularly as safe breeding places may become a trap when predators learn to access them systematically. Because sand goannas, unlike other predators, apparently are able to access nestlings through the narrow nest box entrance (33 mm), and can jump across the gap that we normally leave between a tree and the nest box (or climb up the stake) they have an opportunity free of competition from other predators, such as other bird species, and cats that are largely deterred by the narrow entrance to the nest box (Figure 4). Snakes appear to have been largely excluded as predators of nests in these nest boxes by the difficulty of climbing up the stake on which the box is attached, or the difficulty of reaching across the gap between the bush and box (there are few arboreal snakes in the area).

## 4 CONCLUSIONS

Taken together, this unique documentation of a predation sequence highlights how predation pressure on a bird population may pose a strong selection pressure on birds to breed in synchrony, benefitting from a dilution effect (Brandl et al., 2019, 2021). The fact that zebra finches regularly inspect each other's nests (Brandl et al., 2019) and visit social hangout trees where they are exposed to individual signature vocalisations and juveniles from other nests (Loning, Fragueira, et al., 2023), may allow them to assess predation risk across the population by direct observation and by monitoring the presence (or sudden absence) of known individuals from these hangout bushes. This system thus offers a unique opportunity to better understand the role of predator–prey dynamics and how prey populations may respond to public information and/or increased selective pressures.

The full video is available as VideoS1 on: DOI 10.5281/zenodo.10245991.

## AUTHOR CONTRIBUTIONS

**Marc Naguib:** Conceptualization (equal); formal analysis (supporting); investigation (equal); methodology (equal); project administration (lead); resources (lead); supervision (lead); validation (equal); visualization (equal); writing – original draft (lead); writing – review and editing (equal). **Evelien ter Avest:** Data curation (equal); investigation (equal); methodology (equal). **Chris Tyson:** Data curation (equal); formal analysis (lead); methodology (equal); software (lead); validation (lead); visualization (lead); writing – original draft (supporting); writing – review and editing (supporting). **Martin J. Whiting:** Conceptualization (equal); writing – original draft (equal); writing – review and editing (equal). **Simon C. Griffith:** Conceptualization (lead); investigation (equal); methodology (equal); project administration (lead); resources (lead); supervision (lead); writing – original draft (equal); writing – review and editing (equal). **Hugo Loning:** Conceptualization (lead); data curation (equal); formal analysis (supporting); investigation (lead); methodology (equal); writing – original draft (supporting); writing – review and editing (supporting).

## CONFLICT OF INTEREST STATEMENT

The authors declare no conflict of interest.

## Data Availability

Upon publication, the data will be public on dryad at https://doi.org/10.5061/dryad.cnp5hqcbv. The video is available on Zenodo at: DOI 10.5281/zenodo.10245991 (https://datadryad.org/stash/share/f9BYejPxygGj8eF00unPj1KLNOzEk5KZd3ShcK1d1FA).
